# The dynamic gut microbiota of zoophilic members of the *Anopheles gambiae* complex (Diptera: Culicidae)

**DOI:** 10.1038/s41598-022-05437-y

**Published:** 2022-01-27

**Authors:** Ashmika Singh, Mushal Allam, Stanford Kwenda, Zamantungwa T. H. Khumalo, Arshad Ismail, Shüné V. Oliver

**Affiliations:** 1 Centre for Emerging Zoonotic and Parasitic Diseases, National Institute for Communicable Diseases of the National Health Laboratory Service, Johannesburg, South Africa; 2 Wits Research Institute for Malaria, School of Pathology, Faculty of Health Sciences, University of the Witwatersrand, Johannesburg, South Africa; 3 Sequencing Core Facility, National Institute for Communicable Diseases of the National Health Laboratory Service, Johannesburg, South Africa; 4 Department of Genetics and Genomics, College of Medicine and Health Sciences, United Arab Emirates University, Al Ain, United Arab Emirates; 5 Department of Veterinary Tropical Diseases, Faculty of Veterinary Science, University of Pretoria, Private Bag X04, Onderstepoort, 0110 South Africa

**Keywords:** Microbiology, Microbial communities, Zoology, Entomology

## Abstract

The gut microbiota of mosquitoes plays a critical role in the life history of the animal. There is a growing body of research characterising the gut microbiota of a range of mosquito species, but there is still a paucity of information on some members of the *Anopheles gambiae* complex. In this study, the gut microbiota of four laboratory strains were characterised. SENN (*Anopheles arabiensis*—insecticide susceptible major vector), SENN DDT (*Anopheles arabiensis*—insecticide resistant major vector), MAFUS (*Anopheles merus*—minor vector) and SANGWE (*Anopheles quadriannulatus*—non-vector) were used in this study. The microbiota of fourth instar larvae, 3-day old, 15-day old non-blood fed and 15-day old blood fed females were characterised by MALDI-TOF mass spectroscopy and 16 s rRNA gene sequencing by next generation sequencing. The four strains differed in species richness but not diversity. The major vectors differ in β-diversity from that of the minor and non-vectors. There was no difference in α- or β-diversity in 15 non-blood fed females and 15-day old females that had 3 blood meals before day 15. These differences may be related to a mixture of the effect of insecticide resistance phenotype as well as a potential relationship to vector competence to a limited extent. Bacterial diversity is affected by species and age. There is also a potential relationship between the differences in gut microbiota and capacity to transmit parasites. This genetic background of the mosquitoes, however, play a major role, and must be considered in this relationship.

## Introduction

The *Anopheles gambiae* complex is a group of morphologically identical mosquitoes that differ in their behaviour and ability to transmit the malaria parasite to human hosts^1^. Three members of the *An. gambiae* complex are found in South Africa. The major malaria vector *An. arabiensis*, a minor malaria vector *An. merus* and a non-malaria vector *An. quadriannulatus* are vectors that are all found in Africa within the malaria endemic provinces of South Africa^2^. Currently, the only species implicated in malaria transmission in South Africa is *An. arabiensis*^3^.

*Anopheles arabiensis* is one of the dominant malaria vector species in Sub-Saharan Africa^1,4^. *Anopheles arabiensis* exhibits anthropophilic (human biting), zoophilic (animal biting), endophagic (indoor feeding), exophagic (outdoor feeding), endophilic (indoor resting) and exophilic (outdoor resting) behavioural patterns^1,5,6^.

The members of the *An. gambiae* complex found in South Africa differ from *An. gambiae ss* and *An. coluzzi* which commonly display exophily and exophagy. Yet, they differ markedly in their vector competence^1^. As such, behavioural differences alone cannot account for these differences. There would also be a molecular basis for these differences, particularly in terms of immunological underpinnings.

To highlight an example of the role of immunity, *An. quadriannulatus* can be artificially infected with *Plasmodium falciparum* at low efficiency^7^, even though this is not usually observed in the wild. The prophenoloxidase system of *An. quadriannulatus* is initiated at lower levels than the major vector *An. gambiae*^8^. This highlights the importance of the immunological response to the parasite. An increased understanding of the immunological differences between vectors and non-vectors would inform on future transmission blocking efforts.

A key factor in determining vector competence is the gut microbiota of the mosquito. The microbiota of the midgut in a mosquito plays crucial roles in development, reproduction, immunity, and vector competence^9^. The composition of the midgut varies with the life cycle stage and diet^10^. The larval gut microbiome is crucial for development as gut-sterilised mosquitoes cannot pupate^10–12^. Larvae feed on the contents of the surrounding water as well as drink the surrounding water. This suggests that the diet obtained from the environment is important in the survival and replacement of new bacteria in the midgut^13,14^.

For malaria to be transmitted by an *Anopheles* mosquito, an infectious blood meal must be ingested and make it through the developmental bottlenecks in the midgut and salivary glands. The midgut is the first and main bottleneck of parasite development, as this is where the parasite undergoes the sexual stages of reproduction. The success of the escape of the motile ookinetes from the midgut is crucial for successful parasite proliferation^15^. Therefore, the study of the dynamics of the midgut of the mosquito is a key field of study to develop alternative vector control strategies.

The midgut microbiota of the *Anopheles* mosquito contributes to the ability of the mosquito to contract and transmit the *Plasmodium* parasite as it is involved with the immunity of the mosquito^16,17^. The midgut microbiota protects the mosquito against *Plasmodium* infection through various pathways. The bacteria in the midgut can act as a physical barrier after a blood meal has been ingested, preventing penetration of the parasite into the midgut wall^18^. The bacteria in the midgut can directly affect the parasite by producing antimicrobial compounds or by inducing oxidative stress. In addition to these other defence mechanisms, the bacteria can activate the NF-κB dependent Immune-deficiency (imd) pathway to fight *Plasmodium* infection^15^.

A search of the term “*Anopheles* microbiome” on Pubmed has increased from 3 records in 2010 to 23 in 2020. This indicates a slow but steady increase of the information in this field. Although the field of mosquito microbiome studies are advancing, the studies tend to be limited in the species studies, typically focussing on *An. stephensi*, *An. coluzzi* and *An. gambiae ss*, examples of studies includes Sharma et al.; Galeano-Castañeda et al.; Saab et al.^17,19,20^. Studies of the microbiome of *An. arabiensis* and other members of the *An. gambiae* complex are notably lacking. Characterising the gut microbiota of *Plasmodium* refractory species could provide information on the role of gut microbiota in this phenotype. This study aimed to characterise the dynamic gut microbiota of the minor vector *An. merus* and the non-vector *An. quadriannulatus*. Furthermore, this will be compared to the dynamic gut microbiome of insecticide resistant and susceptible *An. arabiensis*.

## Results

### Culture-dependent characterisation of the dynamic gut microbiota by MALDI-TOF MS

The species identified by MALDI-TOF MS were dominated by Gram-negative bacteria, specifically γ-proteobacteria of the family *Enterobacteriaceae* (0.41). The remaining γ-proteobacteria were from the families of *Yersiniaceae* (0.09), *Aeromonadaceae*, (0.08). *Pseudomonadaceae* (0.07), *Moraxellaceae* (0.05), and *Morganellaceae* (0.02). The next most abundant classes were the *Bacilli* (0.02) and *Actinobacteria* (0.01), which constituted the majority of the Gram-positive bacteria identified. The final significant Gram-negative class was the *Weeksellaceae* (0.08) family. The results of the MALDI-TOF study are summarised in Supplementary Table S1.

### Culture-independent characterisation of the dynamic gut microbiota by 16S rRNA gene sequencing

#### Alpha diversity determined by 16S sequencing

**Figure 1:**
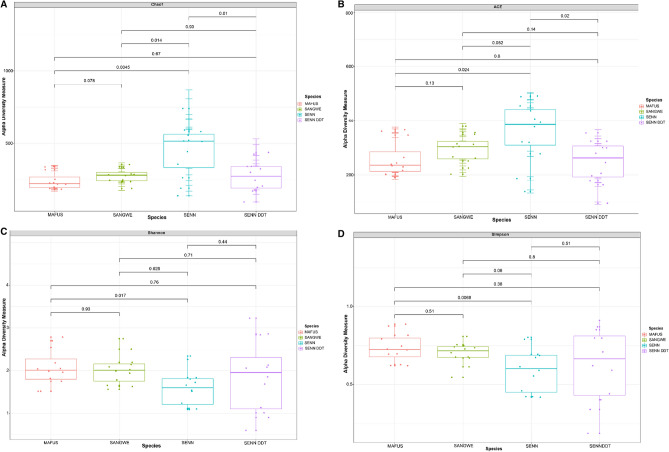
Comparison of alpha diversity between the zoophilic members of the *An. gambiae* complex. (**A**) Chao1 index. (**B**) Abundance-based Coverage Estimator (ACE) index. (**C**) Shannon diversity index. (**D**) Simpson diversity index.

When comparing the alpha diversity of the complete groups, the species richness indices (a measure of the total number of species in the relevant strain and treatment) Chao1 and ACE (summarised in Table 2) indicate a significant difference between the strains. Species evenness indicators (a measure of relative abundance of species in the relevant strain and treatment), Shannon and Simpson (summarised in Table 3), do not indicate a significant difference. Chao 1 indices indicated a significant difference between strains (Kruskal–Wallis One-way ANOVA: p < 0.01). SENN (*An. arabiensi*s-insecticide susceptible) had a significantly higher index than SENN DDT (*An. arabiensis*-insecticide resistant) (p = 0.01), as well as SANGWE (*An. quadriannulatus*) (p = 0.01) and MAFUS (*An. merus*) (p < 0.01) (Fig. 1A). Similarly, with ACE indicators, there was a significant difference between the strains (Kruskal–Wallis One-way ANOVA: p = 0.03). SENN had a significantly higher ACE index than SENN-DDT (p = 0.02). Although SENN did not have a significantly different ACE index than MAFUS (p = 0.05), it did have a significantly higher ACE index than SANGWE (p = 0.02). MAFUS and SANGWE did not differ in ACE indices (p = 0.13) (Fig. 1B).

Overall, the Shannon diversity indices of the strains did not differ (Kruskal–Wallis One-way ANOVA: p = 0.14). SANGWE had a significantly higher Shannon diversity index than SENN (p = 0.03) as did MAFUS (p = 0.02). SENN DDT did not differ significantly from SENN (p = 0.44) (Fig. 1C). The Simpson’s diversity index was also not significantly different (Kruskal–Wallis One-way ANOVA: p = 0.14). The only significant difference in Simpson’s index was significantly higher indices in MAFUS than SENN DDT (p < 0.01) (Fig. 1D).

When comparing alpha diversity between the life stages of the strains, the Chao 1 index of the fourth instar larvae was significantly higher than that of 15-day old sugar fed adults (p < 0.01) and 15-day old blood fed adults (p = 0.02). The ACE index of the fourth instar larvae was significantly higher than that of 3-day old adults (p = 0.02), 15-day old non-blood fed adults (p < 0.01), and 15-day old blood fed adults (p < 0.01). The Shannon index of the larvae was significantly higher than that of 3-day old adults (p < 0.01), 15-day old non-blood fed adults (p < 0.01) and 15-day old blood fed adults (p < 0.01). The Simpson diversity index of the larvae was significantly higher than that of 3-day old adults (p < 0.01), 15-day old non-blood fed adults (p < 0.01) and 15-day old blood fed adults (p < 0.01). There was no difference in species richness between older blood fed and non-blood fed adults as Chao 1 indices did not differ significant (p = 0.71) neither did ACE indices differ (p = 0.63). This was also true for species evenness, as there were no significant differences in Shannon diversity indices (p = 0.41) or Simpson diversity indices (p = 0.93) (Supplementary Table S2).

**Figure 2:**
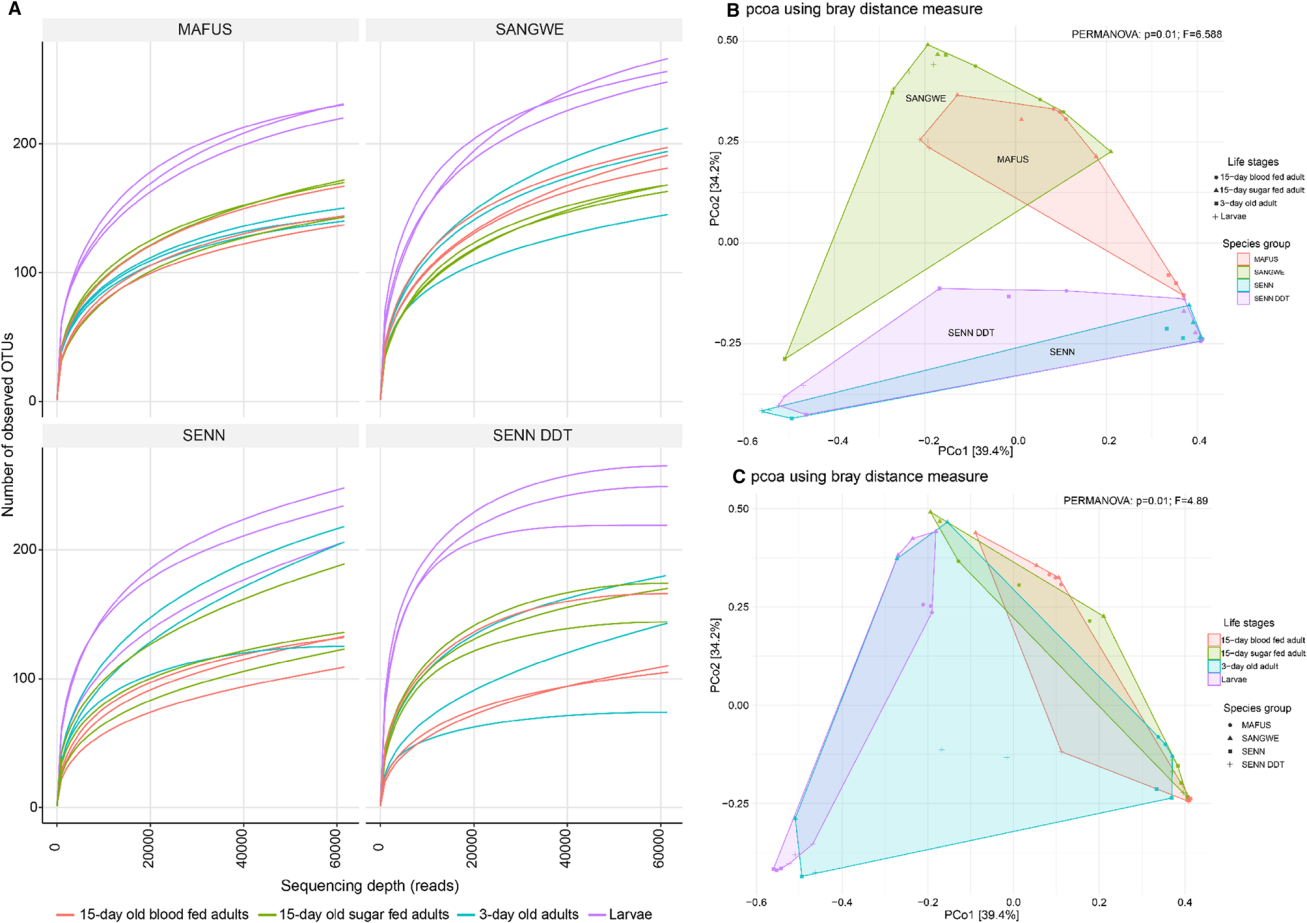
Rarefaction curves and beta diversity plots of the *An. gambiae* complex. (**A**) rarefaction curves of MAFUS (*An. merus*), SANGWE (*An. quadriannulatus*), SENN (*An. arabiensis*-insecticide susceptible) and SENN-DDT (*An. arabiensis*-insecticide resistant). (**B**) Principle Coordinates Analysis plot of beta-diversity highlighting the differences between strains. (**C**) Principal Component Analysis plot of beta-diversity highlighting differences between life stages.

Within the strains, only MAFUS had a significant difference within Chao1 index between the life stages (Kruskal–Wallis One-way ANOVA: p = 0.03). There was no difference in ACE indices between the life stages of SENN (p = 0.31), SENN DDT (p = 0.44), MAFUS (p = 0.05) and SANGWE (p = 0.07). When examining indices examining evenness rather than the richness, there was a significant difference in Shannon diversity indices between life stages in SANGWE (p = 0.03) as well as MAFUS (p = 0.03), but not in SENN (p = 0.09) or SENN DDT (p = 0.08). When examining Simpson diversity indices, only SENN had a significant difference in indices between life stages (p = 0.04) (Supplementary Table S2). The variation in the findings is confirmed by rarefaction analysis, with larval diversity being the greatest in all samples. This analysis also confirms the depth of sequencing (Fig. 2A). Alpha diversities indices are summarised in Tables 1 and 2.

**Table 1:** Chao-1 and ACE diversity indexes of the bacteria located in the midgut at the different life stages of female members of the *Anopheles gambiae* complex for 16S rRNA sequencing.

	SENN *An. arabiensis*	SENN DDT *An. arabiensis*	*An. merus*	*An. quadriannulatus*	SENN *An. arabiensis*	SENN DDT *An. arabiensis*	*An. merus*	*An. quadriannulatus*
	Chao1 diversity indices				ACE diversity indices			
Fourth instar larvae	151	115	114	179	160	115	111	127
3-day adults	65.1	84.6	75.4	91.4	67.7	85.9	74.1	93.7
15-day old non-blood fed adults	105	62.2	74.4	97.7	104	63.3	70.5	94.7
15-day old blood fed adults	83.1	82.9	71.6	103	71.9	69.6	71.3	130

**Table 2:** Shannon and Simpson diversity indexes of the bacteria located in the midgut at the different life stages of female members of the *Anopheles gambiae* complex for 16S rRNA sequencing.

	SENN *An. arabiensis*	SENN DDT *An. arabiensis*	*An. merus*	*An. quadriannulatus*	SENN *An. arabiensis*	SENN DDT *An. arabiensis*	*An. merus*	*An. quadriannulatus*
	Shannon diversity indices				Simpson diversity indices			
Fourth instar larvae	2.05	2.90	2.62	2.37	0.896	0.754	0.859	0.738
3-day adults	1.65	1.16	1.93	1.91	0.438	0.650	0.681	0.702
15-day old non-blood fed adults	1.44	1.83	1.92	1.61	0.660	0.518	0.743	0.615
15-day old blood fed adults	1.15	1.41	1.65	2.00	0.5482	0.451	0.660	0.741

#### Beta diversity analysis determined by 16S sequencing

Two β-diversity analyses were performed, and both confirmed the same patterns. NMDS analysis demonstrated that MAFUS (*An. merus*) and SANGWE (*An. quadriannulatus*) clustered together and were significantly different from SENN (*An. arabiensis*-insecticide susceptible) and SENN DDT (*An. arabiensi*s-insecticide resistant) (PERMANOVA: p = 0.01, F = 6.59) (Fig. 2B). Principle Co-ordinates analysis (PCoA) also confirmed this finding (PERMANOVA: p = 0.01, F = 6.59). There was also a significant difference in β-diversity of larvae and 15-day old adults, with 3-day old females overlapping the different age groups (PERMANOVA: p = 0.01, F = 4.89) (Fig. 2C).

### Comparison of bacterial species overlaps and differential abundance in mosquito strains

**Figure 3:**
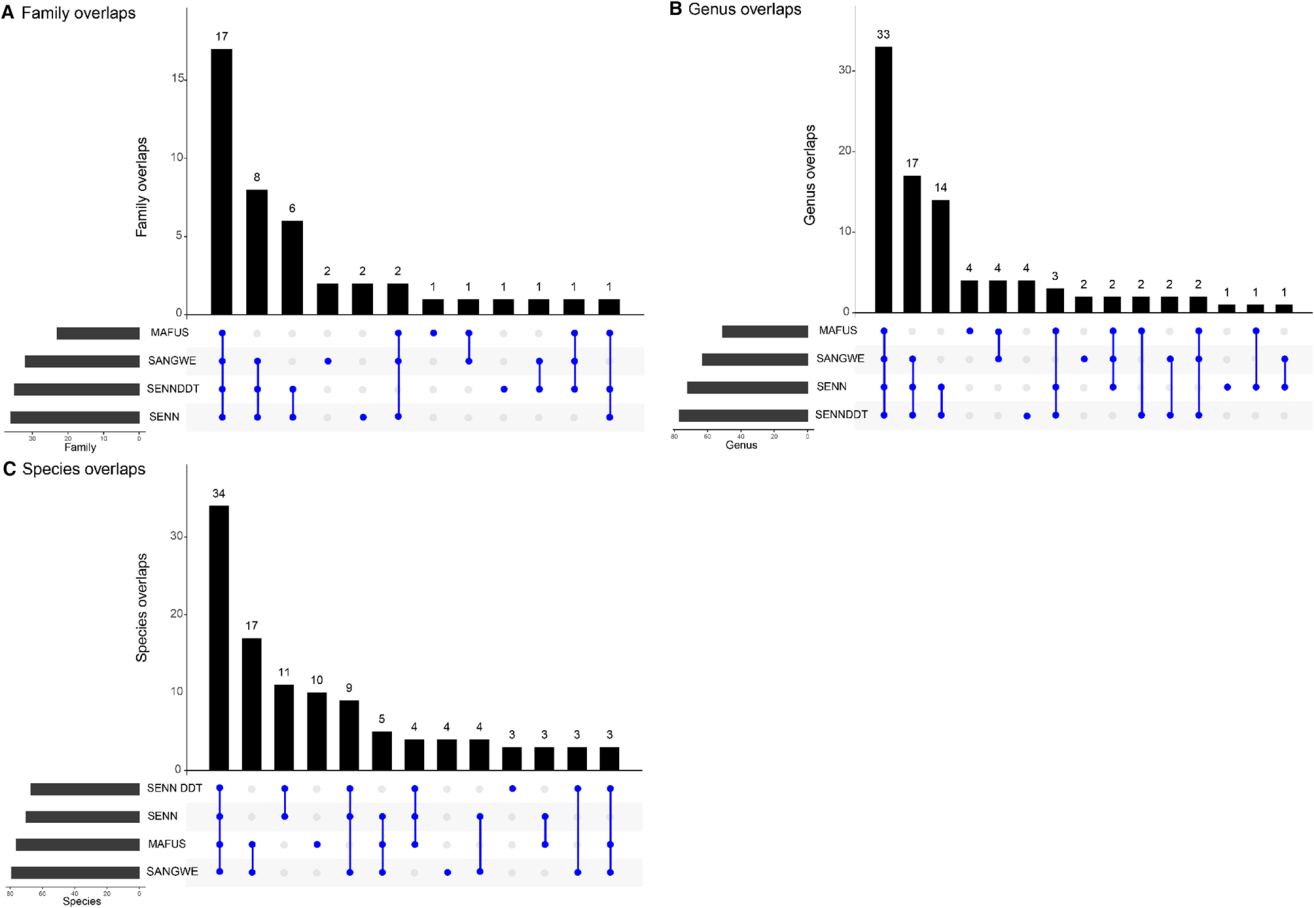
Overlapping midgut bacteria identified between the different mosquito strains. The blue dots represent the number of overlaps per strain, with single dots indicating the number of unique families/genera/species per strain. (**A**) Comparison of common families. There are 17 common families between all four strains. (**B**) Comparison of common genera. There are 33 common genera between all four strains. (**C**) Comparison of common species. There are 34 common species between all strains.

When examining the overlap in bacterial specimens identified, all four strains had 17 families in common. SENN (*An. arabiensis*-insecticide susceptible) and SENN DDT (*An. arabiensi*s-insecticide resistant) shared 6 families, while SANGWE (*An. quadriannulatus*) and MAFUS (*An. merus*) shared only one family (Fig. 3A). When examining overlapping genera, all four strains had 33 genera in common. MAFUS and SENN DDT had the most unique genera with four each. There were 14 shared genera between SENN and SENN DDT, but only 4 shared between MAFUS and SANGWE (Fig. 3B). When examining species composition, MAFUS had 10 unique species, the most of any of the strains. SANGWE had 4 unique species, and SENN DDT had 3 unique species. SENN had no unique species present (Fig. 3C).

Differential abundance was assessed between strains. For each pairwise comparison, genera fold changes with adjusted p-values (q-value) < 0.01 were considered differentially abundant. When comparing SENN to SANGWE *Aeromonas* and *Aquitalea* had the greatest fold increased abundance in SENN, and *Mesorhizobium* and *Sphingopyxis* having the smallest fold change. Notable abundance differences were increased *Elizabethkingia* and *Rhanella* in SENN and increased *Pseudomonas*, *Klebsiella* and *Serratia* in SANGWE. Similarly, *Aquitalea* and *Aeromonas* had the greatest fold increased abundance in SENN DDT compared to SANGWE, and *Mesorhizobium* and *Microbacterium* having the smallest fold change. Notable abundance differences were higher abundances of *Rhanella* and *Elizabethkingia* in SENN-DDT, and increased abundances of *Pseudomonas* and *Klebsiella* in SANGWE.

**Figure 4:**
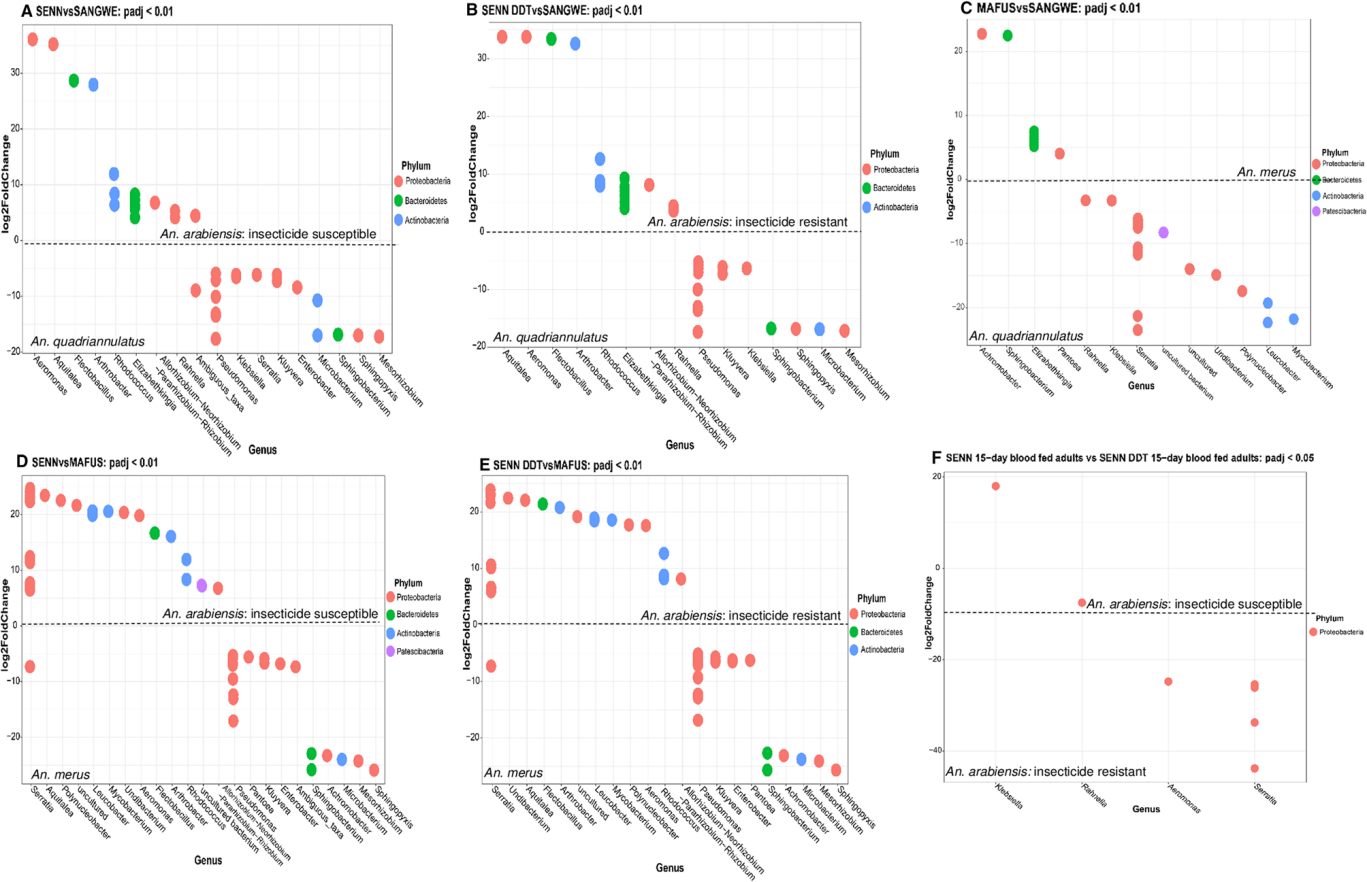
Comparison of differential abundance of genera between strains. (**A**) Differential abundance of genera in SENN vs SANGWE at an 99% Confidence interval. (**B**) Differential abundance of genera in SENN-DDT vs SANGWE at a 99% Confidence interval. (**C**) Differential abundance of genera in MAFUS vs SANGWE at a 99% Confidence interval. (**D**) Differential abundance of genera in SENN vs MAFUS at a 99% Confidence interval. (**E**) Differential abundance of genera in SENN vs SANGWE at an 99% Confidence interval. (**F**) Differential abundance of genera in 15-day bloodfed SENN vs 15 -day bloodfed SENN-DDT at a 95% confidence interval. No genus was found to be significantly abundantly expressed between these two strains overall at the 99% confidence interval. The dotted line at 0 represents the change from over representation to underrepresentation of bacterial genera. The species name on either side of the dotted line where the relevant bacterial species are abundant.. Each dot represents a single OTU.

When examining differential abundance between MAFUS and the two *An. arabiensis* strains, SENN and SENN DDT, *Serratia* was the genus with the greatest differential abundance in both SENN and SENN DDT compared to MAFUS. The smallest differential abundance in both SENN and SENN DDT compared to MAFUS are *Sphingopyxis* and *Mesorhizobium*. *Pseudomonas* and *Enterobacter* were more differentially abundant in MAFUS than either SENN or SENN DDT. When comparing the differential abundance of MAFUS and SANGWE, *Acromobacter*, *Sphingobacterium* and *Elizabethkingia* had the greatest differential abundance in MAFUS. *Mycobacterium*, *Leucobacterium* and *Polynucleobacter* had the least differential abundance in MAFUS. There were no genera differentially abundant between SENN and SENN DDT at q-value < 0.01 significance level. When using a less stringent significance level of q-value < 0.05, differentially abundant species were largely observed when comparing blood-fed females. *Klebsiella* were more abundant in SENN, while *Rhanella*, *Aeromonas* and *Serratia* were more abundant in SENN DDT (Fig. 4). With the same adjustment, *Cedecea*, *Klebsiella*, *Enterobacter* and *Rhanella* had a greater differential abundance in 3-day old SENN and *Zooglea* had the lowest differential abundance. The fold changes associated with the differential abundance are summarised in Tables 3 and 4.

**Table 3:** Statistical indicators for differential abundance of OTUs at a 99% confidence interval.

	SENN (*An. Arabiensis*—insecticide susceptible)	SENN-DDT (*An. Arabiensis*—insecticide resistant)	MAFUS (*An. merus* )	SANGWE (*An. quadriannulatus* )
SENN (*An. arabiensis*—insecticide susceptible)		None	*Sphingopyxis* (Log2 fold change: − 25.90) *Mesorhizobium* (Log2 fold change: − 24.23) *Pseudomonas* (Log2 fold change: − 17.04) *Enterobacter* (Log2 fold change: − 6.79)	*Mesorhizobium* (Log2 fold change: − 17.29) *Sphingopyxis* (log fold change: − 16.96) *Pseudomonas* (Log2 fold change: − 13.61) *Klebsiella* (Log2 fold change: − 6.52) *Serratia* (Log2 fold change: − 6.12)
SENN-DDT (*An. arabiensis*—insecticide resistant)	None		*Sphingopyxis* (Log2 fold change: − 25.69) *Mesorhizobium* (Log2 fold change: − 24.03) *Pseudomonas* (Log2 fold change: − 16.91) *Enterobacter* (Log2 fold change: − 6.52)	*Mesorhizobium* (Log2 fold change: − 17.09) *Microbacterium* (Log2 fold change: − 16.87) *Pseudomonas* (Log2 fold change: − 17.04) *Elizabethkingia* (Log2 fold change: − 9.10) *Klebsiella* (Log2 fold change: − 5.03)
MAFUS (*An. merus* )	*Serratia* (Log2 fold change: 24.48)	*Serratia* (Log2 fold change: 23.94)		*Mycobacterium* (Log2 fold change: − 21.91 *Leucobacterium* (Log2 fold change: − 19.27) *Polynucleobacter* (Log2 fold change: − 17.46)
SANGWE (*An. quadriannulatus* )	*Aeromonas* (Log2 fold change: 38.02) *Aquitalea* (Log2 fold change: 35.20) *Elizabethkingia* (Log2 fold change: 8.25) *Rhanella* (Log2 fold change: 5.31)	*Aquitalea* (Log2 fold change: 33.79) *Aeromonas* (Log2 fold change: 33.76) *Rhanella* (Log2 fold change: 4.30)	*Acromobacter* (Log2 fold change: 22.74) *Sphingobacterium* (Log2 fold change: 22.47) *Elizabethkingia* (Log2 fold change: 7.35)	

The differential abundance is given for the overall life stages. The strain at the top is compared to the strain on the right, with the fold change referring to the change relevant to the strain at the top.

**Table 4:** Statistical indicators for differential abundance between SENN and SENN-DDT at a 95% confidence interval.

	SENN (*An. arabiensis*—insecticide susceptible) 15-day-blood	SENN-DDT (*An. arabiensis*—insecticide resistant) 15-day-blood
SENN (*An. arabiensis*-insecticide susceptible) 15 day blood		*Rhanella* (Log2 fold change: − 7.54) *Aeromonas* (Log2 fold change: − 24.79) *Serratia* (Log2 fold change: − 43.82)
SENN-DDT (*An. arabiensis*-insecticide resistant) 15 day blood	*Klebsiella* (Log2 fold change: 17.98)	

	SENN (*An. arabiensis*—insecticide susceptible) 3-day-old	SENN-DDT (*An. arabiensis*—insecticide resistant) 3-day-old
SENN (*An. arabiensis*-insecticide susceptible)		*Zooglea* (Log2 fold change: -24.86)
SENN-DDT (*An. arabiensis*-insecticide resistant)	*Cedecea* (Log2 fold change: 8.95)	
	*Klebsiella* (Log2 fold change: 5.01)	
	*Enterobacter* (Log2 fold change: − 17.46)	
	*Rhanella* (Log2 fold change: 4.48)	

The differential abundances specifically compare relative abundance of OTUs at specific life stages as indicated.

## Discussion

The amount of mosquito species where the gut microbiota is being characterised is increasing^12,21–23^ and as such the catalogue of microbiome information available for comparison is increasing. The use of MALDI-TOF MS resulted in a markedly less species identified than NGS. These findings were congruent with a recent finding that analysed the microbiota of laboratory and wild *An. arabiensis* by MALDI-TOF MS where similar numbers and species were identified^21^. For the MS technique to be used for larger scale microbiome studies, culture protocols will have to be optimised. This will include optimising the choice of culture media as well as growing plates under anaerobic conditions to maximise the diversity of samples cultured. The use of an alternate database could also potentially improve the quality of analysis. Therefore, although less data was obtained by MALDI-TOF analysis, it is still a viable technique for identifying mosquito gut microbiota.

NGS was the more sensitive technique. This technique, however, are more prone to contamination which can result in misidentification. A common source of contamination is often the DNA extraction kit. In support of this, a study by Mancini et al.^24^ demonstrated that some of the most common species associated with kit-borne contamination found in mosquito microbiome study included *Mesorhizobium*, *Phyllobacterium*, *Rhizobium*, *Comamonas*, *Delftia*, *Variovorax* and *Escherichia*-*Shigella*^24^. In the current study *Phyllobacterium* and *Rhizobium* were not identified, while *Mesorhizobium*, *Comamonas*, *Delftia*, *Variovorax* and *Escherichia*-*Shigella* were detected at low abundance (< 0.01% prevalence). The human skin contaminants *Corynebacterium* (12 OTUs) and *Streptococcus* (119 OTUs) were also identified at low abundance (< 0.01% prevalence), while *Propionibacterium*, another skin-associated contaminant was not identified at all.

There have been several studies associating gut microbiota with insecticide resistance^22,23,25^. These studies compared the gut microbiota of fenitrothion resistant and susceptible *An. albimanus* populations^23^ as well as observing distinct gut microbiota in pyrethroid resistance in *An. gambiae*^22^. The insecticide susceptible SENN strain had a greater species richness than the insecticide resistant SENN-DDT strain, but this did not translate into a difference in species diversity. Both strains also had overlapping β-diversity. There were also no bacterial species differentially abundant in the strains at the 99% confidence, unlike when compared to other strains. The increased number of species in SENN suggests a reduction in bacterial species due to selection for resistance^23^. However, the presence of species and genera associated with insecticide degradation in both species suggests that this is not a significant contributor to insecticide resistance in the SENN DDT strain. As the SENN DDT strain is fixed for the L1014F mutation as well as having elevated detoxification enzymes^26^, it is likely that the contribution of insecticide degrading bacteria to the resistant phenotype is minimal. It is, however, worth noting that *Sphingobacterium*, an insecticide degrading genus^22^, was significantly reduced in the insecticide susceptible SANGWE. A previous study found *Microbacterium* was unique to insecticide susceptible *An. albimanus*^23^. In this study, *Microbacterium* had a greater relative abundance in the insecticide susceptible SANGWE and MAFUS, which has low level insecticide tolerance.

Regardless of strains, larvae had the greatest species richness and diversity of all the life stages. This is congruent with previous findings on the dynamic gut microbiota of *An. gambiae*^12^, where species richness decreased with age and blood feeding status. The shift in microbiota between the aquatic and aerial stages has been observed previously^10,27^. Furthermore, in an examination of the dynamic gut microbiota of *An. gambiae*, diversity decreased with age and blood feeding status^12,28^. This was confirmed by comparing the differential abundance of the combined groups, where larvae had nineteen genera with a higher differential abundance compared to six in 15-day old females which had multiple blood meals. *Asaia*, *Elizabethkingia*, *Serratia* and *Rhanella* were more abundant in blood fed females, while *Aquitalea*, *Sphingobacterium* and *Sphiyngopyxis* were the most abundantly expressed in larvae. Consistent with previous findings, this represents a shift from aerobic to facultatively anaerobic^29^. Unlike other studies, this study did not examine the direct effect of blood on gut microbiota. Rather, it examined the longer-term effects of multiple blood meals. This is because multiple blood meals are a common feeding strategy (e.g., Norris et al.)^30^ which is known to increase the *Plasmodium* infectivity^31^. The lack of species richness and diversity in the adult stages examined in this study suggests that specific bacterial species, rather than population changes contribute to the microbial effect on life history.

A range of factors affect the composition of the gut microbiota. This includes the rearing water, food and sugar source^20^. Like with previous studies, several common species constituted the majority of gut microflora. These include *Enterobacter*, *Rhanella*, *Klebsiella*, *Serratia*, *Pseudomonas*, *Elizabethkingia*, *Asaia* and *Raoultella*. This supports the suggestion of a core gut microbiota. A core microbiome has been suggested to exist for *An. gambiae* and *An. coluzzi*^32^. As the gut microbiome is markedly less diverse than that of the salivary glands and reproductive organs^24,33^, it is plausible that a set of bacteria could constitute the core gut microbiota of anophelines. It has also been suggested that the core microbiota persists over time^34^, which is also supported by this study.

There have not been many studies that have characterised members of the *An. gambiae* complex. The majority of studies have focused on the major vector *An. gambiae* (e.g., Gimonneau et al.; Boissière et al.)^33,35^. There are fewer examples of *An. arabiensis* to compare to (E’Silva et al.; Barnard et al.)^21,36^ and even less for *An. quadriannulatus* and *An. merus*^24^. As such, there are not many databases for to compare the data generated in this study. A notable conservation is that in *An. arabiensis* from the E’Silva study, which constitutes young, unfed females are dominated by *Serratia* as are both *An. arabiensis* strains in this study. Interestingly, this congruency does not hold true for the dominance of *Pseudomonas* in *An. merus* and *An. quadriannulatus* when compared to Mancini et al.^24^.

The amount of microbiome data available for members of the An. gambiae complex outside that of the nominal member^33^ is relatively limited. A single previous study examined the microbiota of several tissues in nine mosquito species, including *An. gambiae*, *An. arabiensis*, *An. merus* and *An. quadriannulatus*^24^. The present study is more similar to Wang et al.^12^ which examined the dynamic gut microbiota of *An. arabiensis*. Although this took place in laboratory strains, the uniformity in larval feeding, sugar feeding and provision of blood meals should limit the rate of false discovery, this cannot be fully guaranteed^20^.

Indices that estimate richness (Chao1 and ACE) indicate a significant difference between the two *An. arabiensis* strains with the insecticide susceptible strain having a significantly higher species richness (number of bacteria). This difference did not extend to species diversity, as determined by Shannon and Simpson indices. *Anopheles merus* and *An. quadriannulatus* did not differ in α-diversity but had higher Shannon indices than the insecticide susceptible *An. arabiensis*. Despite the lack of difference in alpha diversity, there is a marked difference in beta diversity between the strains, with the major vector species SENN and SENN DDT clustering separately from the minor vector MAFUS and the non-vector species SANGWE. This is markedly different to the lack of beta diversity between wild *An. gambiae* and *An. coluzzi*^33^. Laboratory reared mosquitoes have been described as having lower gut microbial diversity than their wild counterparts (as reviewed in Dada et al.)^37^. Despite this, a significant difference in beta diversity was observed in the present study.

*Anopheles gambiae* and *An. coluzzi* are both efficient major vectors of malaria that do not differ in microbial beta diversity. By contrast, *An. arabiensis*, *An. merus* and *An. quadriannulatus* differ in vector competence. This difference in vector competence is unlikely to be explained fully by behaviour. *Anopheles arabiensis*, *An. merus* and *An. quadriannulatus* are all primarily zoophilic and outdoor biting. Therefore, the differences in vector competence may have further molecular underpinnings, such as variable immune responses (e.g., Habtewold et al.)^38^. The differences in gut microbiota between the major, minor, and non-vectors may therefore contribute vector competence, although this needs to be confirmed by *Plasmodium*-infection studies.

Due to their anti-*Plasmodium* activities, *Asaia*, *Pantoea*, *Pseudomonas*, *Enterobacter*, *Serratia* are gaining considerable attention as promising candidates for paratransgenic modifications for vector control strategies^24^. *Pseudomonas putida*, *Pantoea sp* and *S. marcescens* were capable of blocking *Plasmodium* development in vivo when introduced as a sugar meal. *Comamonas* sp., *Acinetobacter sp*., *P. putida* and *P. rhodesiae*, *Pantoea sp*, *S. marcescens* and *E. anophelis* demonstrated *Plasmodium* blocking activity in vitro^39^. The *Enterobacter* isolate Esp_Z inhibits *Plasmodium* development from gametocyte to ookinete by in vivo and in vitro production of reactive oxygen species^40^. When comparing differential abundance of *An. quadriannulatus* to both *An. arabiensis* strains, *Pseudomonas* was more abundant in the former. Additionally, *Serratia* and *Enterobacter* was more abundant in *An. quadriannulatus* compared to the insecticide susceptible *An. arabiensis*. *Pseudomonas*, *Pantoea*, and *Enterobacter* had a greater differential abundance in *An. meus*. This further suggests that differential gut microbiota may contribute to differences in vector competence between the strains *Serratia* had a higher differential abundance in *An. quadriannulatus*. *Serratia*, however, had a greater differential abundance in the *An. arabiensis* strains. It is worth noting, however, that despite the association of *Serratia* with anti-*Plasmodium* defense, this is strain dependent^41^. *Serratia marcescens*, however, is known to degrade several insecticides^23^. The higher expression in the *An. arabiensis* strains may be related more to insecticide resistance than anti-parasite defense.

The microbiota of *An. merus* and *An. quadriannulatus* is poorly examined. Although this study does contribute information to this small pool, there are several limitations to this study. The first limitation is that this study requires replication on field specimens (as described in Romoli and Gendrin)^15^ Secondly, experiments need to be performed examining the effect of *Plasmodium* infection on gut microbial diversity to confirm the species-specific role of microbiota in vector competence. It would also be worth examining changes in the microbiota of the salivary glands after infection. Furthermore, it is worth considering the effect of saltwater breeding on the microbiota of *An. merus*. As an example, *Rhanella* is a freshwater bacterial species^42^, potentially explaining the low expression of this species in *An. merus*.

The *An. merus* strain was reared in salt water, but as an osmotolerant species, considerations must be made of the effect of salt concentration on the gut microbiota of this species. The variation in beta diversity between the two *An. arabiensis* strains and the *An. merus* and *An. quadriannulatus* may be due to the evolutionary distance between the species, but the lack of beta diversity between *An. quadriannulatus* and *An. merus* should be taken into account as motivation for the validity of these findings. Although the findings could suggest a role for gut microbiota in differences in *Plasmodium* transmission, these findings must be replicated with wild mosquitoes, and preferably with experiments that examine the changes in gut microbiota before and after *Plasmodium* infection. These experiments are, however, beyond the scope of this study.

In conclusion, this study characterised the dynamic gut microbiota of *An. merus*, *An. quadriannulatus* and *An. arabiensis* by culture-dependent and culture independent techniques. Although α-diversity did not differ greatly, there was a significant difference in β-diversity of the major vector *An. arabiensis* and minor vector *An. merus* and the non-vector *An. quadriannulatus*. This diversity, in conjunction with the decreased relative abundance of genera associated with anti-*Plasmodium* effects in *An. arabiensis* suggests that this difference may be associated with vector competence. Although both the insecticide susceptible and resistant *An. arabiensis* harboured insecticide degrading bacteria, genera associated with insecticide susceptibility was associated with the insecticide susceptible *An. quadriannulatus* strain. Therefore, this study suggests a role for differential microbial diversity in the life history of zoophilic members of the *An. gambiae* complex.

## Materials and methods

### Mosquito collection and preparation for characterisation

Laboratory reared *Anopheles* mosquitoes used were housed and collected from the Botha de Meillon insectary of the National Institute of Communicable Disease (NICD), Johannesburg, South Africa. Four strains, representing three species were used in this study. The *An. arabiensis* strain SENN is an insecticide susceptible strain originated in Sennar, Sudan. SENN has been in colony since 1980. The *An. arabiensis* strain SENN DDT is an insecticide resistant strain that has continuously been selected for DDT resistance from the SENN strain since 1995. This strain is still being selected for DDT resistance. SENN DDT is fixed for the *kdr* L1014F mutation. It also has elevated cytochrome P450, general esterase as well as Glutathione S-transferase activity. This accounts for the strain’s resistance to DDT, permethrin, deltamethrin, λ-cyhalothrin and malathion^26,43,44^. The *An. quadriannulatus* strain (SANGWE) is insecticide susceptible and originated from Zimbabwe. MAFUS is an *An. merus* strain originating from Malahlapanga, South Africa and is tolerant to insecticides. It has been in colony since 2012 and was reared in 50% seawater.

Larvae were reared in reverse osmosis water at 25 °C (± 2 °C), 80% relative humidity (± 5%) with a 12-h light/dark cycle and a 30-min dusk/dawn cycle^45^. Larvae were fed a mixture of powder Beano™ dog biscuits and yeast. Several life stages of *An. arabiensis* (SENN and SENN DDT), *An. merus* and *An. quadriannulatus* were collected: fourth instar larvae, 3-day old female adults, 15-day old non-blood fed female adults and 15-day old blood fed female adults. Blood feeding took place at the age of 3, 7 and 11 days. Blood was supplied by a single consenting human volunteer, the author Shüné V. Oliver, as per the ethics waiver provided by the University of the Witwatersrand (Ethics waiver number: 09-06-2020-O). Blood was supplied as a live feeding volunteer, and no other individuals besides the author were involved in providing the blood meal. For MALDI-TOF mass spectrometric analysis of the midgut microbiota, 180 female mosquitoes and 60 larvae were used. For 16S rRNA gene sequencing analysis of the midgut microbiota, 180 female mosquitoes and 60 larvae were used.

### Matrix assisted laser desorption ionisation-time of flight (MALDI-TOF) mass spectrometric analysis of the midgut microbiota

#### Midgut resection and culturing procedure

Larvae were chilled at 4 °C to minimise their activity and adult female mosquitoes were cold killed at − 70 °C for 5 min prior to midgut resection. Strict sterile conditions were implemented, the forceps, microscope, microscope slides and mosquitoes were sterilised with 70% ethanol. The midguts from the respective samples were dissected using a dissection microscope (Olympus, SZ2-ILST) with a magnification of 40×. Five replicates consisting of three midguts each were suspended in 200 µl of 0.1 M Phosphate Buffered Saline (PBS) pH 7.2.

The extracted guts were aseptically added to 2 ml of Lysogeny Broth (LB broth). The culturing tubes were incubated at 37 °C overnight with constant shaking at approximately 120 rpm. The negative control consisted of PBS in uninoculated LB broth. Each of the samples were plated onto three types of media, namely, Brain Heart Infusion BHI agar (for the culture of fastidious organisms), a 10% blood agar (for the culture of fastidious organisms) and a MacConkey agar (selective for lactose fermenters). All culture plates were obtained from Diagnostic Media Products (South Africa). Single colonies were obtained by streak plating with subsequent incubation at 37 °C for 18 h.

#### Bacterial identification using MALDI-TOF spectroscopy

The mass spectrometry system used in this study is housed in the Centre for Healthcare-Associated Infections, Antimicrobial Resistance and Mycoses (CHARM) at the National Institute of Communicable Diseases in Johannesburg, South Africa.

To perform the analysis individual bacterial colonies were selected manually from each plate based on the size, colour, growth pattern or shape and transparency. The individual colony was selected and applied to a 96-well MALDI target plate (Bruker Daltonics, Wissembourg, France; cat no. 8280800) in duplicate. Brucker Matrix HCCA (α-Cyano-4-hydroxycinnamic acid), portioned at 2.5 ± 0.3 mg matrix (Hain Life Sciences) was dissolved in 250 µl Standard solvent (acetonitrile 50%, water 47.5% and trifluoroacetic acid 2.5%) (Sigma-Aldrich). One microlitre of Bruker HCCA Bruker (10 mg Bruker HCCA/mL) was added to each occupied target spot position and left to dry at room temperature.

The target plate was placed into the Brucker Matrix-assisted Laser Desorption Ionising Biotyper System with a benchtop Microflex LT/SH mass spectrometer (Bruker Daltonics, Wissembourg, France). The bacteria were identified on 16 s rRNA spectra as per manufacturer’s instructions. For each spectrum obtained a maximum of 100 peaks were used and compared with other peaks in the MBT 7854 MSP Library (Bruker Daltonics, Wissembourg, France; ref no. 182903). The bacteria were accepted as correctly identified if the identification score was ≥ 1.9^46^.

For all downstream analysis α-diversity indices were divide into species richness and species evenness indicators. The Chao1 and ACE indices were used to measure species richness, a direct count of the Operational Taxonomic Units (OTUs). The Shannon and Simpson diversity indices were used as measures of species evenness. The Shannon and Simpson diversity index is a measure of diversity which considers both richness as well as their relative abundance or evenness.

### 16S rRNA gene sequencing analysis of the midgut microbiota

#### Midgut dissection and DNA extraction

The midguts of the female adult mosquitoes and larvae were dissected as previously mentioned however, five replicates consisting of five midguts each were suspended in 200 µl of 0.1 M PBS pH 7.2. To ensure sterile conditions, all equipment and surfaces used were sterilised using 70% ethanol and 3% hydrogen peroxide. An extraction negative control of 0.1 M PBS pH 7.2 only was used during the course of the DNA extraction.

Total genomic DNA from the midgut of the mosquito was extracted using the DNeasy PowerSoil^®^ Kit (QIAGEN: 12888-50). The tubes containing the midguts were centrifuged at 5000×*g* and approximately 150 µl of PBS was discarded, the remaining PBS and midguts were vortexed briefly and added to the PowerBead™ tubes. The protocol was then followed as per manufacturer’s instructions.

#### Amplification of the V3-V4 hypervariable region of the 16S rRNA gene

The V3-V4 hypervariable region of the bacterial 16S rRNA gene was amplified using universal bacterial primers. Forward Primer (5′ GTC TCG TGG GCT CGG AGA TGT GTA TAA GAG ACA GGA CTA CHV GGG TAT CTA ATC C 3′)(Integrated DNA Technologies) and Reverse Primer (5′ TCG TCG GCA GCG TCA GAT GTG TAT AAG AGA CAG CCT ACG GGN GGC WGC AG 3′) (4 nmol Ultramer^®^ DNA Oligo 55 bases : Integrated DNA Technologies)^47^. Library preparation was performed according to the standard instructions of the 16S Metagenomic Sequencing Library Preparation protocol (IlluminaTM, Inc., San Diego, CA, United States). The size of the amplicons were then visualised using the 4200 TapeStation (Agilent Technologies). Two negative controls were used in the amplicon preparation step. The extraction negative control as well as no template control were used in the initial amplification step. No amplification was observed in either of the negative controls.

Libraries were then sequenced on the MiSeq platform using MiSeq Reagent kit v3 (Illumina) and paired-end 2 × 300 bp sequencing was performed at the Sequencing Core Facility, National Institute for Communicable Diseases, South Africa.

#### Bioinformatic analysis

QIAGEN CLC Genomics Workbench 20 (CLC Bio Qiagen) was used to identify the operational taxonomic units (OTU’s) of each of the specimens analysed. Amplicon-based analysis workflows were used for data quality control and OTU clustering.

Sequences were filtered, trimmed and subjected to quality control to ensure clean data was used for clustering. The raw reads were trimmed with a 0.05 quality limit. Clustering of the data was done at a 99% similarity threshold with the SILVA 16S v132 database. The OTU’s were assigned to sequences based on 97% identity. The OTU table was then used to obtain a visual representation of the midgut bacteria of each respective sample in the form of a bar plot.

Bacterial diversity analysis was done using raw reads, which were quality controlled and filtered (Q > 20 and length > 50 bp) using fastqc (v0.11.8) and trimGalore (v0.6.4_dev; https://github.com/FelixKrueger/TrimGalore), respectively^48,49^. In addition, trimGalore was used for adapter removal.

All the downstream analyses were performed in R (v3.6.1), including classification, abundance estimations, statistical analysis, and visualisation. Clean reads were pre-processed using dada2 package (v1.12.1)^50^, including quality inspection, trimming, dereplication, merging paired-end reads and removal of chimeric sequences.

Taxonomy was assigned to the obtained amplicon sequence variants (ASVs) and the ASV abundance estimates determined using training sequence sets based on the SILVA reference database (v138; https://zenodo.org/record/1172783#.XvCmtkUzY2w).

Ordinations for beta diversity, abundance bar plots, alpha diversity and richness estimates, and heatmaps were generated using the phyloseq package (v1.28.0)^51^, ggplot2 (v3.2.1) and AmpVis2 package (v2.6.4)^52^. Data clustering in NMDS were assessed using PERMANOVA (permutation test with pseudo-F ratios) as implemented in the adonis function in the vegan package (https://github.com/vegandevs/vegan).

Kruskal–Wallis rank-sum tests were used to compare alpha diversity between groups. Venn diagrams were generated using Venn Diagram (v1.6.20) and UpsetR (v1.4.0)^53^. Differential abundance analysis between sample groups was performed using DESeq2 (v1.24.0)^54^.

## Supplementary Information

Supplementary Figure S1.

Supplementary Figure S2.

Supplementary Table S1.

Supplementary Table S2.

Supplementary Table S3.

Supplementary Table S4.
