# Supplementary material for: The dynamic gut microbiota of zoophilic members of the Anopheles gambiae complex (Diptera: Culicidae)
Source: Sci Rep. 2022 Jan 27;12:1495. doi: 10.1038/s41598-022-05437-y (PMC8795440; doi:10.1038/s41598-022-05437-y)
Supplement: Supplementary file 1 — Supplementary Figure S1. [file 41598_2022_5437_MOESM1_ESM.pdf]

Supplementary Figure 1: MALDI-TOF MS results

Characterisation of the dynamic gut microbiota of primarily zoophilic members of the *Anopheles gambiae* complex (Diptera: Culicidae).

Ashmika Singh[1,2], Mushal Allam[3,4], Stanford Kwenda[3], Zamantungwa T.H. Khumalo[3,5], Arshad Ismail[3], Shüné V. Oliver [1,2*]

1: Centre for Emerging Zoonotic and Parasitic Diseases, National Institute for Communicable Diseases of the National Health Laboratory Service, Johannesburg, South Africa

2: Wits Research Institute for Malaria, School of Pathology, Faculty of Health Sciences, University of the Witwatersrand, Johannesburg, South Africa.

3: Sequencing Core Facility, National Institute for Communicable Diseases of the National Health Laboratory Service, Johannesburg, South Africa

4: Department of Genetics and Genomics, College of Medicine and Health Sciences, United Arab Emirates University, United Arab Emirates.

5: Department of Veterinary Tropical Diseases, Faculty of Veterinary Science, University of Pretoria, Private Bag X04, Onderstepoort, 0110, South Africa.

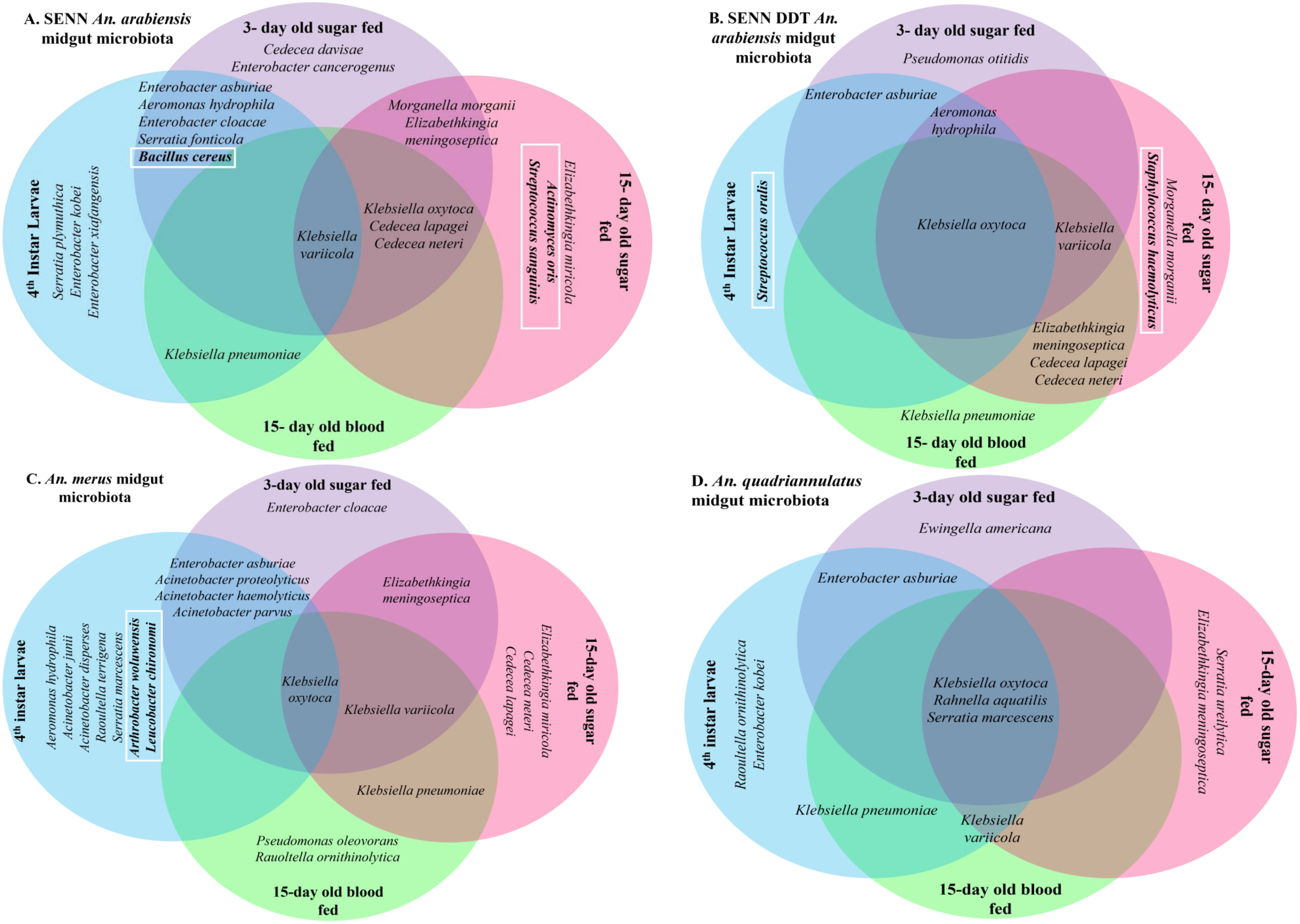

A. SENN An. arabiensis midgut microbiota
3- day old sugar fed
Cedecea davisae
Enterobacter cancerogenus
Enterobacter asburiae
Aeromonas hydrophila
Enterobacter cloacae
Serratia fonticola
Bacillus cereus
Morganella morganii
Elizabethkingia meningoseptica
4th Instar Larvae
Serratia plymuthica
Enterobacter kobei
Enterobacter xiafangensis
Klebsiella variicola
Klebsiella oxytoca
Cedecea lapagei
Cedecea neteri
Streptococcus sanguinis
Actinomyces oris
Elizabethkingia miricola
15- day old sugar fed
Klebsiella pneumoniae
15- day old blood fed
B. SENN DDT An. arabiensis midgut microbiota
3- day old sugar fed
Pseudomonas otitidis
Enterobacter asburiae
Aeromonas hydrophila
4th Instar Larvae
Streptococcus oralis
Klebsiella oxytoca
Klebsiella variicola
Staphylococcus haemolyticus
Morganella morganii
15- day old sugar fed
Elizabethkingia meningoseptica
Cedecea lapagei
Cedecea neteri
Klebsiella pneumoniae
15- day old blood fed
C. An. merus midgut microbiota
3-day old sugar fed
Enterobacter cloacae
Enterobacter asburiae
Acinetobacter proteolyticus
Acinetobacter haemolyticus
Acinetobacter parvus
Elizabethkingia meningoseptica
4th instar larvae
Aeromonas hydrophila
Acinetobacter junii
Acinetobacter disperses
Raoultella terrigena
Serratia marcescens
Arthrobacter woluwensis
Leucobacter chironomi
Klebsiella oxytoca
Klebsiella variicola
Cedecea lapagei
Cedecea neteri
Elizabethkingia miricola
15-day old sugar fed
Klebsiella pneumoniae
Pseudomonas oleovorans
Rauoltella ornithinolytica
15-day old blood fed
D. An. quadriannulatus midgut microbiota
3-day old sugar fed
Ewingella americana
Enterobacter asburiae
4th instar larvae
Raoultella ornithinolytica
Enterobacter kobei
Klebsiella oxytoca
Rahnella aquatilis
Serratia marcescens
Elizabethkingia meningoseptica
Serratia ureilytica
15-day old sugar fed
Klebsiella pneumoniae
Klebsiella variicola
15-day old blood fed

Supplementary Figure 1: Venn diagrams of species identified by MALDI-TOF MS across the lifespan of zoophilic members of the *An. gambiae* complex. Bacterial species identified in the midgut of 4th instar larvae, 3-day old sugar fed females, 15-day old non-blood fed females and 15-day old females. Gram positive bacteria are highlighted with a white block.
