# Supplementary material for: The dynamic gut microbiota of zoophilic members of the Anopheles gambiae complex (Diptera: Culicidae)
Source: Sci Rep. 2022 Jan 27;12:1495. doi: 10.1038/s41598-022-05437-y (PMC8795440; doi:10.1038/s41598-022-05437-y)
Supplement: Supplementary file 2 — Supplementary Figure S2. [file 41598_2022_5437_MOESM2_ESM.pdf]

# Characterisation of the dynamic gut microbiota of primarily zoophilic members of the *Anopheles gambiae* complex (Diptera: Culicidae).

**Ashmika Singh[1,2], Mushal Allam[3,4], Stanford Kwenda[3], Zamantungwa T.H. Khumalo[3,5], Arshad Ismail[3], Shüné V. Oliver [1,2*]**

1: Centre for Emerging Zoonotic and Parasitic Diseases, National Institute for Communicable Diseases of the National Health Laboratory Service, Johannesburg, South Africa

2: Wits Research Institute for Malaria, School of Pathology, Faculty of Health Sciences, University of the Witwatersrand, Johannesburg, South Africa.

3: Sequencing Core Facility, National Institute for Communicable Diseases of the National Health Laboratory Service, Johannesburg, South Africa

4: Department of Genetics and Genomics, College of Medicine and Health Sciences, United Arab Emirates University, United Arab Emirates.

5: Department of Veterinary Tropical Diseases, Faculty of Veterinary Science, University of Pretoria, Private Bag X04, Onderstepoort, 0110, South Africa.

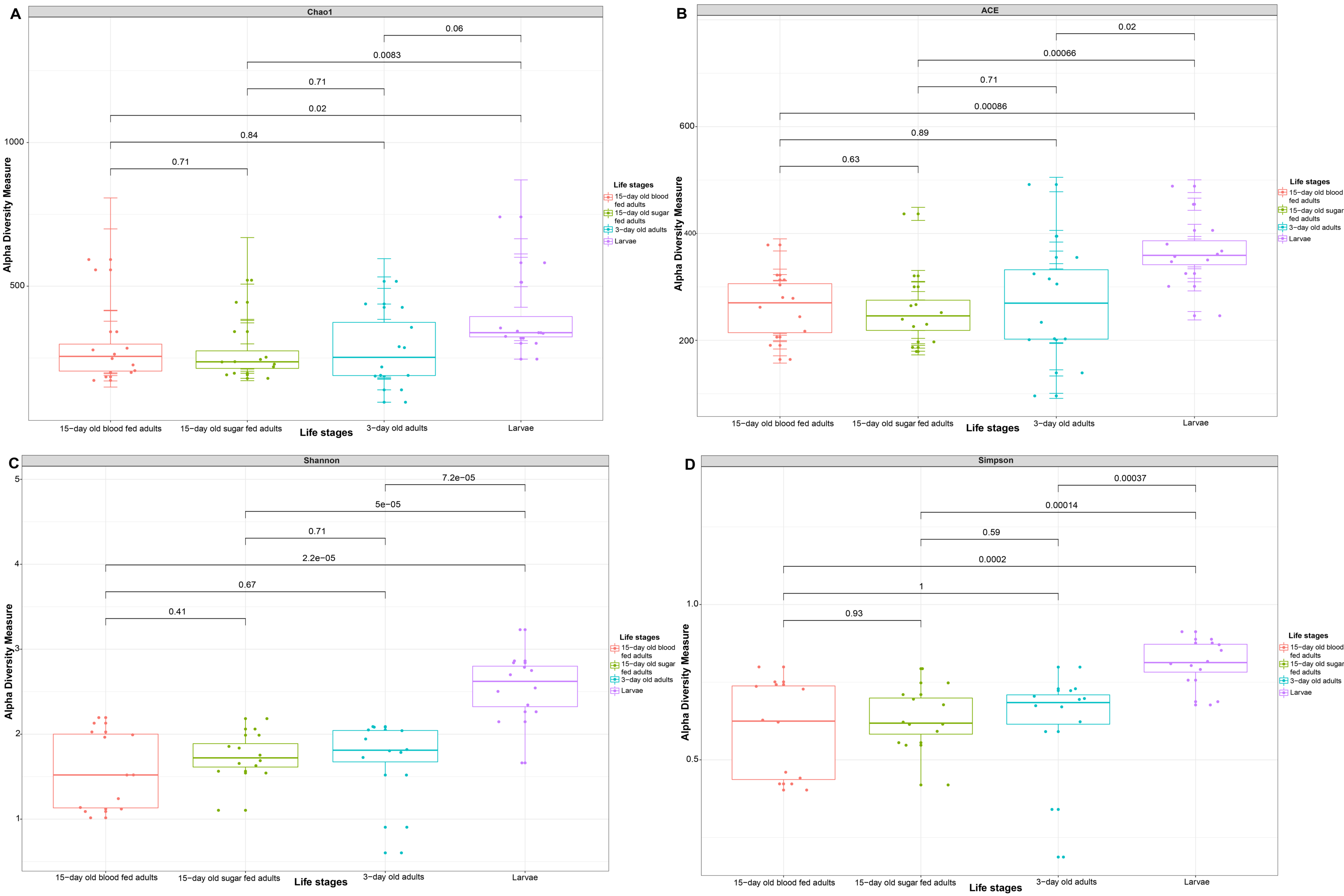

A
Chao1
0.06
0.0083
0.71
0.02
0.84
0.71
1000
500
Alpha Diversity Measure
Life stages
15−day old blood fed adults
15−day old sugar fed adults
3−day old adults
Larvae
B
ACE
0.02
0.00066
0.71
0.00086
0.89
0.63
600
400
200
C
Shannon
7.2e−05
5e−05
0.71
2.2e−05
0.67
0.41
5
4
3
2
1
D
Simpson
0.00037
0.00014
0.59
0.0002
1
0.93
1.0
0.5

**Supplementary Figure 1: Comparison of alpha diversity between the life stages of zoophilic members of the *An. gambiae* complex.** A: Chao1 index. B: Abundance-based Coverage Estimator (ACE) index. C: Shannon diversity index. D: Simpson diversity index.

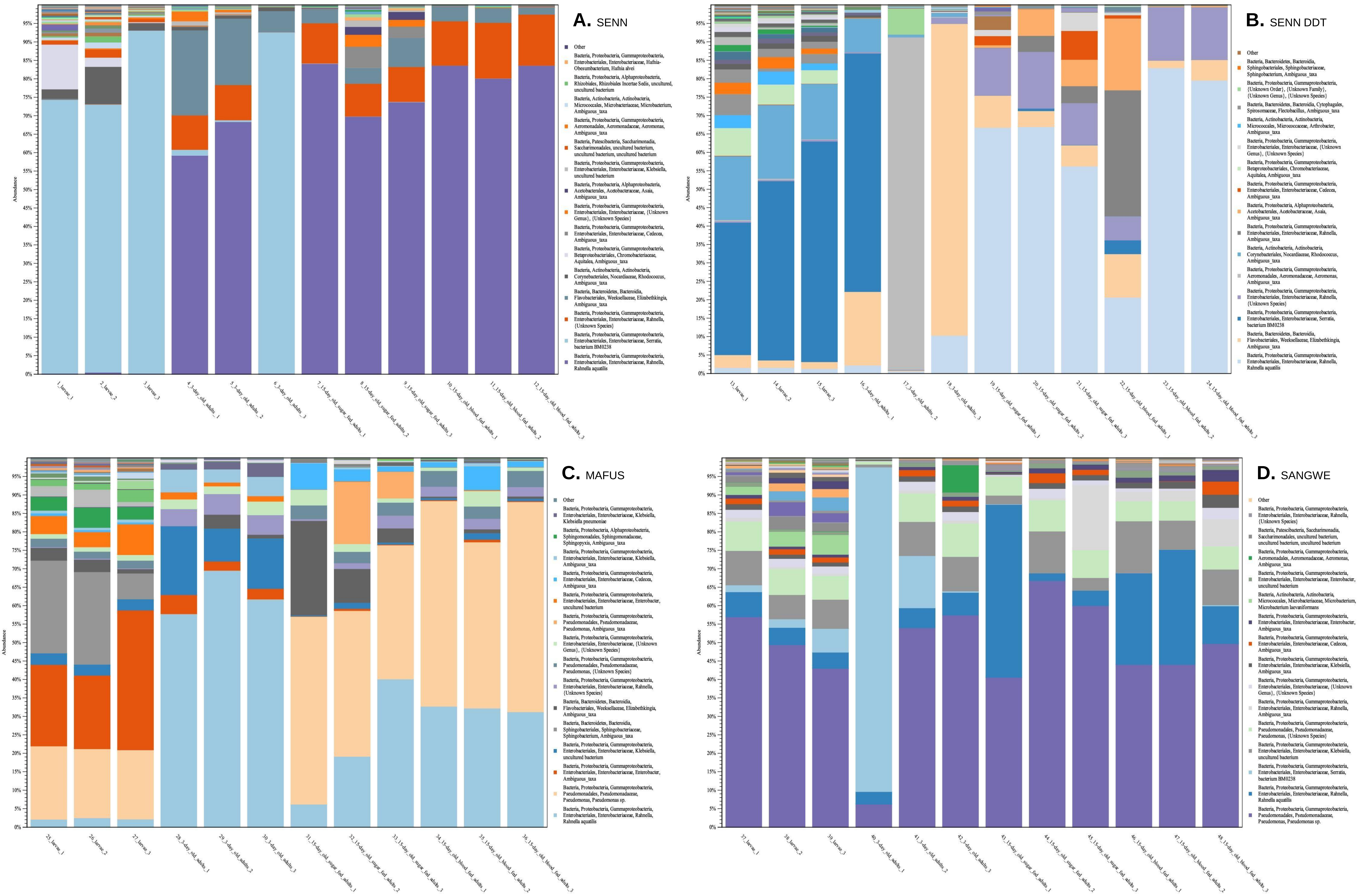

A. SENN
B. SENN DDT
C. MAFUS
D. SANGWE
Abundance
0%
5%
10%
15%
20%
25%
30%
35%
40%
45%
50%
55%
60%
65%
70%
75%
80%
85%
90%
95%
1_larvae_1
2_larvae_2
3_larvae_3
4_3-day_old_adults_1
5_3-day_old_adults_2
6_3-day_old_adults_3
7_15-day_old_sugar_fed_adults_1
8_15-day_old_sugar_fed_adults_2
9_15-day_old_sugar_fed_adults_3
10_15-day_old_blood_fed_adults_1
11_15-day_old_blood_fed_adults_2
12_15-day_old_blood_fed_adults_3
Other
Bacteria, Proteobacteria, Gammaproteobacteria, Enterobacteriales, Enterobacteriaceae, Hafnia-Obesumbacterium, Hafnia alvei
Bacteria, Proteobacteria, Alphaproteobacteria, Rhizobiales, Rhizobiales Incertae Sedis, uncultured, uncultured bacterium
Bacteria, Actinobacteria, Actinobacteria, Micrococcales, Microbacteriaceae, Microbacterium, Ambiguous_taxa
Bacteria, Proteobacteria, Gammaproteobacteria, Aeromonadales, Aeromonadaceae, Aeromonas, Ambiguous_taxa
Bacteria, Patescibacteria, Saccharimonadia, Saccharimonadales, uncultured bacterium, uncultured bacterium, uncultured bacterium
Bacteria, Proteobacteria, Gammaproteobacteria, Enterobacteriales, Enterobacteriaceae, Klebsiella, uncultured bacterium
Bacteria, Proteobacteria, Alphaproteobacteria, Acetobacterales, Acetobacteraceae, Asaia, Ambiguous_taxa
Bacteria, Proteobacteria, Gammaproteobacteria, Enterobacteriales, Enterobacteriaceae, {Unknown Genus}, {Unknown Species}
Bacteria, Proteobacteria, Gammaproteobacteria, Enterobacteriales, Enterobacteriaceae, Cedecea, Ambiguous_taxa
Bacteria, Proteobacteria, Gammaproteobacteria, Betaproteobacteriales, Chromobacteriaceae, Aquitalea, Ambiguous_taxa
Bacteria, Actinobacteria, Actinobacteria, Corynebacteriales, Nocardiaceae, Rhodococcus, Ambiguous_taxa
Bacteria, Bacteroidetes, Bacteroidia, Flavobacteriales, Weeksellaceae, Elizabethkingia, Ambiguous_taxa
Bacteria, Proteobacteria, Gammaproteobacteria, Enterobacteriales, Enterobacteriaceae, Rahnella, {Unknown Species}
Bacteria, Proteobacteria, Gammaproteobacteria, Enterobacteriales, Enterobacteriaceae, Serratia, bacterium BM0238
Bacteria, Proteobacteria, Gammaproteobacteria, Enterobacteriales, Enterobacteriaceae, Rahnella, Rahnella aquatilis
13_larvae_1
14_larvae_2
15_larvae_3
16_3-day_old_adults_1
17_3-day_old_adults_2
18_3-day_old_adults_3
19_15-day_old_sugar_fed_adults_1
20_15-day_old_sugar_fed_adults_2
21_15-day_old_sugar_fed_adults_3
22_15-day_old_blood_fed_adults_1
23_15-day_old_blood_fed_adults_2
24_15-day_old_blood_fed_adults_3
Other
Bacteria, Bacteroidetes, Bacteroidia, Sphingobacteriales, Sphingobacteriaceae, Sphingobacterium, Ambiguous_taxa
Bacteria, Proteobacteria, Gammaproteobacteria, {Unknown Order}, {Unknown Family}, {Unknown Genus}, {Unknown Species}
Bacteria, Bacteroidetes, Bacteroidia, Cytophagales, Spirosomaceae, Flectobacillus, Ambiguous_taxa
Bacteria, Actinobacteria, Actinobacteria, Micrococcales, Micrococcaceae, Arthrobacter, Ambiguous_taxa
Bacteria, Proteobacteria, Gammaproteobacteria, Enterobacteriales, Enterobacteriaceae, {Unknown Genus}, {Unknown Species}
Bacteria, Proteobacteria, Gammaproteobacteria, Betaproteobacteriales, Chromobacteriaceae, Aquitalea, Ambiguous_taxa
Bacteria, Proteobacteria, Gammaproteobacteria, Enterobacteriales, Enterobacteriaceae, Cedecea, Ambiguous_taxa
Bacteria, Proteobacteria, Alphaproteobacteria, Acetobacterales, Acetobacteraceae, Asaia, Ambiguous_taxa
Bacteria, Proteobacteria, Gammaproteobacteria, Enterobacteriales, Enterobacteriaceae, Rahnella, Ambiguous_taxa
Bacteria, Actinobacteria, Actinobacteria, Corynebacteriales, Nocardiaceae, Rhodococcus, Ambiguous_taxa
Bacteria, Proteobacteria, Gammaproteobacteria, Aeromonadales, Aeromonadaceae, Aeromonas, Ambiguous_taxa
Bacteria, Proteobacteria, Gammaproteobacteria, Enterobacteriales, Enterobacteriaceae, Rahnella, {Unknown Species}
Bacteria, Proteobacteria, Gammaproteobacteria, Enterobacteriales, Enterobacteriaceae, Serratia, bacterium BM0238
Bacteria, Bacteroidetes, Bacteroidia, Flavobacteriales, Weeksellaceae, Elizabethkingia, Ambiguous_taxa
Bacteria, Proteobacteria, Gammaproteobacteria, Enterobacteriales, Enterobacteriaceae, Rahnella, Rahnella aquatilis
25_larvae_1
26_larvae_2
27_larvae_3
28_3-day_old_adults_1
29_3-day_old_adults_2
30_3-day_old_adults_3
31_15-day_old_sugar_fed_adults_1
32_15-day_old_sugar_fed_adults_2
33_15-day_old_sugar_fed_adults_3
34_15-day_old_blood_fed_adults_1
35_15-day_old_blood_fed_adults_2
36_15-day_old_blood_fed_adults_3
Other
Bacteria, Proteobacteria, Gammaproteobacteria, Enterobacteriales, Enterobacteriaceae, Klebsiella, Klebsiella pneumoniae
Bacteria, Proteobacteria, Alphaproteobacteria, Sphingomonadales, Sphingomonadaceae, Sphingopyxis, Ambiguous_taxa
Bacteria, Proteobacteria, Gammaproteobacteria, Enterobacteriales, Enterobacteriaceae, Klebsiella, Ambiguous_taxa
Bacteria, Proteobacteria, Gammaproteobacteria, Enterobacteriales, Enterobacteriaceae, Cedecea, Ambiguous_taxa
Bacteria, Proteobacteria, Gammaproteobacteria, Enterobacteriales, Enterobacteriaceae, Enterobacter, uncultured bacterium
Bacteria, Proteobacteria, Gammaproteobacteria, Pseudomonadales, Pseudomonadaceae, Pseudomonas, Ambiguous_taxa
Bacteria, Proteobacteria, Gammaproteobacteria, Enterobacteriales, Enterobacteriaceae, {Unknown Genus}, {Unknown Species}
Bacteria, Proteobacteria, Gammaproteobacteria, Pseudomonadales, Pseudomonadaceae, Pseudomonas, {Unknown Species}
Bacteria, Proteobacteria, Gammaproteobacteria, Enterobacteriales, Enterobacteriaceae, Rahnella, {Unknown Species}
Bacteria, Bacteroidetes, Bacteroidia, Flavobacteriales, Weeksellaceae, Elizabethkingia, Ambiguous_taxa
Bacteria, Bacteroidetes, Bacteroidia, Sphingobacteriales, Sphingobacteriaceae, Sphingobacterium, Ambiguous_taxa
Bacteria, Proteobacteria, Gammaproteobacteria, Enterobacteriales, Enterobacteriaceae, Klebsiella, uncultured bacterium
Bacteria, Proteobacteria, Gammaproteobacteria, Enterobacteriales, Enterobacteriaceae, Enterobacter, Ambiguous_taxa
Bacteria, Proteobacteria, Gammaproteobacteria, Pseudomonadales, Pseudomonadaceae, Pseudomonas, Pseudomonas sp.
Bacteria, Proteobacteria, Gammaproteobacteria, Enterobacteriales, Enterobacteriaceae, Rahnella, Rahnella aquatilis
37_larvae_1
38_larvae_2
39_larvae_3
40_3-day_old_adults_1
41_3-day_old_adults_2
42_3-day_old_adults_3
43_15-day_old_sugar_fed_adults_1
44_15-day_old_sugar_fed_adults_2
45_15-day_old_sugar_fed_adults_3
46_15-day_old_blood_fed_adults_1
47_15-day_old_blood_fed_adults_2
48_15-day_old_blood_fed_adults_3
Other
Bacteria, Proteobacteria, Gammaproteobacteria, Enterobacteriales, Enterobacteriaceae, Rahnella, {Unknown Species}
Bacteria, Patescibacteria, Saccharimonadia, Saccharimonadales, uncultured bacterium, uncultured bacterium, uncultured bacterium
Bacteria, Proteobacteria, Gammaproteobacteria, Aeromonadales, Aeromonadaceae, Aeromonas, Ambiguous_taxa
Bacteria, Proteobacteria, Gammaproteobacteria, Enterobacteriales, Enterobacteriaceae, Enterobacter, uncultured bacterium
Bacteria, Actinobacteria, Actinobacteria, Micrococcales, Microbacteriaceae, Microbacterium, Microbacterium laevaniformans
Bacteria, Proteobacteria, Gammaproteobacteria, Enterobacteriales, Enterobacteriaceae, Enterobacter, Ambiguous_taxa
Bacteria, Proteobacteria, Gammaproteobacteria, Enterobacteriales, Enterobacteriaceae, Cedecea, Ambiguous_taxa
Bacteria, Proteobacteria, Gammaproteobacteria, Enterobacteriales, Enterobacteriaceae, Klebsiella, Ambiguous_taxa
Bacteria, Proteobacteria, Gammaproteobacteria, Enterobacteriales, Enterobacteriaceae, {Unknown Genus}, {Unknown Species}
Bacteria, Proteobacteria, Gammaproteobacteria, Enterobacteriales, Enterobacteriaceae, Rahnella, Ambiguous_taxa
Bacteria, Proteobacteria, Gammaproteobacteria, Pseudomonadales, Pseudomonadaceae, Pseudomonas, {Unknown Species}
Bacteria, Proteobacteria, Gammaproteobacteria, Enterobacteriales, Enterobacteriaceae, Klebsiella, uncultured bacterium
Bacteria, Proteobacteria, Gammaproteobacteria, Enterobacteriales, Enterobacteriaceae, Serratia, bacterium BM0238
Bacteria, Proteobacteria, Gammaproteobacteria, Enterobacteriales, Enterobacteriaceae, Rahnella, Rahnella aquatilis
Bacteria, Proteobacteria, Gammaproteobacteria, Pseudomonadales, Pseudomonadaceae, Pseudomonas, Pseudomonas sp.

**Supplementary Figure 2: Gut bacterial composition of the four strains used in the study at species level.** A: SENN; Insecticide susceptible *Anopheles arabiensis* B: SENN DDT Insecticide resistant *An. arabiensis* C: MAFUS; *An. merus* D: SANGWE: *An. quadriannulatus*.
