# Supplementary material for: The dynamic gut microbiota of zoophilic members of the Anopheles gambiae complex (Diptera: Culicidae)
Source: Sci Rep. 2022 Jan 27;12:1495. doi: 10.1038/s41598-022-05437-y (PMC8795440; doi:10.1038/s41598-022-05437-y)
Supplement: Supplementary file 3 — Supplementary Table S1. [file 41598_2022_5437_MOESM3_ESM.pdf]

Supplementary Table 1. Shannon-diversity indices of the bacteria located in the midgut at the different life stages of female members of the *Anopheles gambiae* complex for MALDI-TOF mass spectrometry results.

	SENN *An. arabiensis*	SENN DDT *An. arabiensis*	*An. merus*	*An. quadriannulatus*
**Fourth instar larvae**	1.99	1.24	2.30	1.81
**3-day adults**	2.40	1.42	1.96	1.42
**15-day old non-blood fed adults**	2.06	1.95	1.80	1.60
**15-day old blood fed adults**	1.42	1.54	1.36	1.51
