# Supplementary material for: The dynamic gut microbiota of zoophilic members of the Anopheles gambiae complex (Diptera: Culicidae)
Source: Sci Rep. 2022 Jan 27;12:1495. doi: 10.1038/s41598-022-05437-y (PMC8795440; doi:10.1038/s41598-022-05437-y)
Supplement: Supplementary file 4 — Supplementary Table S2. [file 41598_2022_5437_MOESM4_ESM.pdf]

Supplementary Table 2. Chao-1 and ACE diversity indexes of the bacteria located in the midgut at the different life stages of female members of the *Anopheles gambiae* complex for 16S rRNA sequencing.

	SENN *An. arabiensis*	SENN DDT *An. arabiensis*	*An. merus*	*An. quadriannulatus*	SENN *An. arabiensis*	SENN DDT *An. arabiensis*	*An. merus*	*An. quadriannulatus*
	Chao1 diversity indices				ACE diversity indices			
Fourth instar larvae	151	115	114	179	160	115	111	127
3-day adults	65.1	84.6	75.4	91.4	67.7	85.9	74.1	93.7
15-day old non-blood fed adults	105	62.2	74.4	97.7	104	63.3	70.5	94.7
15-day old blood fed adults	83.1	82.9	71.6	103	71.9	69.6	71.3	130
